# Run-off election-based decision method for the training and inference process in an artificial neural network

**DOI:** 10.1038/s41598-020-79452-2

**Published:** 2021-01-13

**Authors:** Jingon Jang, Seonghoon Jang, Sanghyeon Choi, Gunuk Wang

**Affiliations:** KU-KIST Graduate School of Converging Science and Technology, Korea University, 145, Anam-ro, Seongbuk-gu, Seoul, 02841 Republic of Korea

**Keywords:** Electronics, photonics and device physics, Electrical and electronic engineering

## Abstract

Generally, the decision rule for classifying unstructured data in an artificial neural network system depends on the sequence results of an activation function determined by vector–matrix multiplication between the input bias signal and the analog synaptic weight quantity of each node in a matrix array. Although a sequence-based decision rule can efficiently extract a common feature in a large data set in a short time, it can occasionally fail to classify similar species because it does not intrinsically consider other quantitative configurations of the activation function that affect the synaptic weight update. In this work, we implemented a simple run-off election-based decision rule via an additional filter evaluation to mitigate the confusion from proximity of output activation functions, enabling the improved training and inference performance of artificial neural network system. Using the filter evaluation selected via the difference among common features of classified images, the recognition accuracy achieved for three types of shoe image data sets reached ~ 82.03%, outperforming the maximum accuracy of ~ 79.23% obtained via the sequence-based decision rule in a fully connected single layer network. This training algorithm with an independent filter can precisely supply the output class in the decision step of the fully connected network.

## Introduction

The artificial neural network (ANN), which consists of a synaptic device unit capable of both processing and memory functions, has attracted great interest as a brain-inspired analog computing architecture that can efficiently address unstructured complex tasks^1–9^. Particularly, the ANN-based computing system for big data processing covers various application ranges, such as for image^10,11^ or voice^12^ recognition, signal spectrum analysis^13,14^, convolution filtering^15^ and use in visual perception systems^16,17^. The conductance modulation capability of a constitutive artificial synaptic node in the ANN can reconfigure the synaptic weight matrix under a learning rule, which is analogous to the essentials of neural plasticity in the human brain. The cumulative output signal is dependent on vector–matrix multiplication using the synaptic weights on each node, which can imitate integrated signal firing through the dendrite of the postneuron in the cortex neural network^18–25^. In this ANN system, the typical decision rule is determined by the sequence of activation function values at each output neuron confined in a fixed range via a monotonic increasing function, such as a sigmoidal or tangent hyperbolic function^26–29^. Based on the magnitude of the fired output values, the classification decision is simply assigned to a certain output neuron that has the highest value. However, this sequence-based decision process is fundamentally limited in its ability to classify data sets in the same object category because it does not consider other configurations of the activation function values. Specifically, when the difference in the activation function values between output neurons is relatively small and the sequence result is incorrectly ordered during training, the ANN system can guide the synaptic weights to the wrong updating direction in a given matrix according to the backpropagation learning rule^30,31^. As a suggested approach for mitigating the abovementioned issue, construction of an auxiliary network in a subsection of the ANN could become an alternative approach that can evaluate the answer among predetermined competitive output neurons in a specified network region at a particular configuration of activation function values. In other words, this approach is based on the run-off election based decision process and is designed to choose a more precise answer between species that possess high scores after the preliminary evaluation.

In this work, we suggest a mutually independent auxiliary synaptic network that can deliver a highly reliable shoe image recognition process in the ANN system by preventing misinterpretations resulting from the conventional decision rule that considers only the sequence of output activation function values. For classification of confusable images, the auxiliary network, which is a quarter of the size of the original pixel image, is partially processed at the assigned filter location selected according to the difference of common features at the preliminary evaluation. In this process, the confusable images are properly reclassified according to the result of the filter evaluation. In other words, the resorting process with the 3-filter auxiliary networks can apply the change in the common feature of the classified output classes and modulation of synaptic weight for the fully connected single layer network (FCSN) which can be determining step in various neural network, leading to a more precise learning direction. As a result, this method can achieve meaningful accuracy improvement for difficult shoe image recognition. This result suggests that filter evaluation based on the run-off election method in the determining step of the ANN system can improve the efficiency of the complex image classification process.

## Methods

### Conventional neural network for the 3-class shoe image inference process

**Figure 1:**
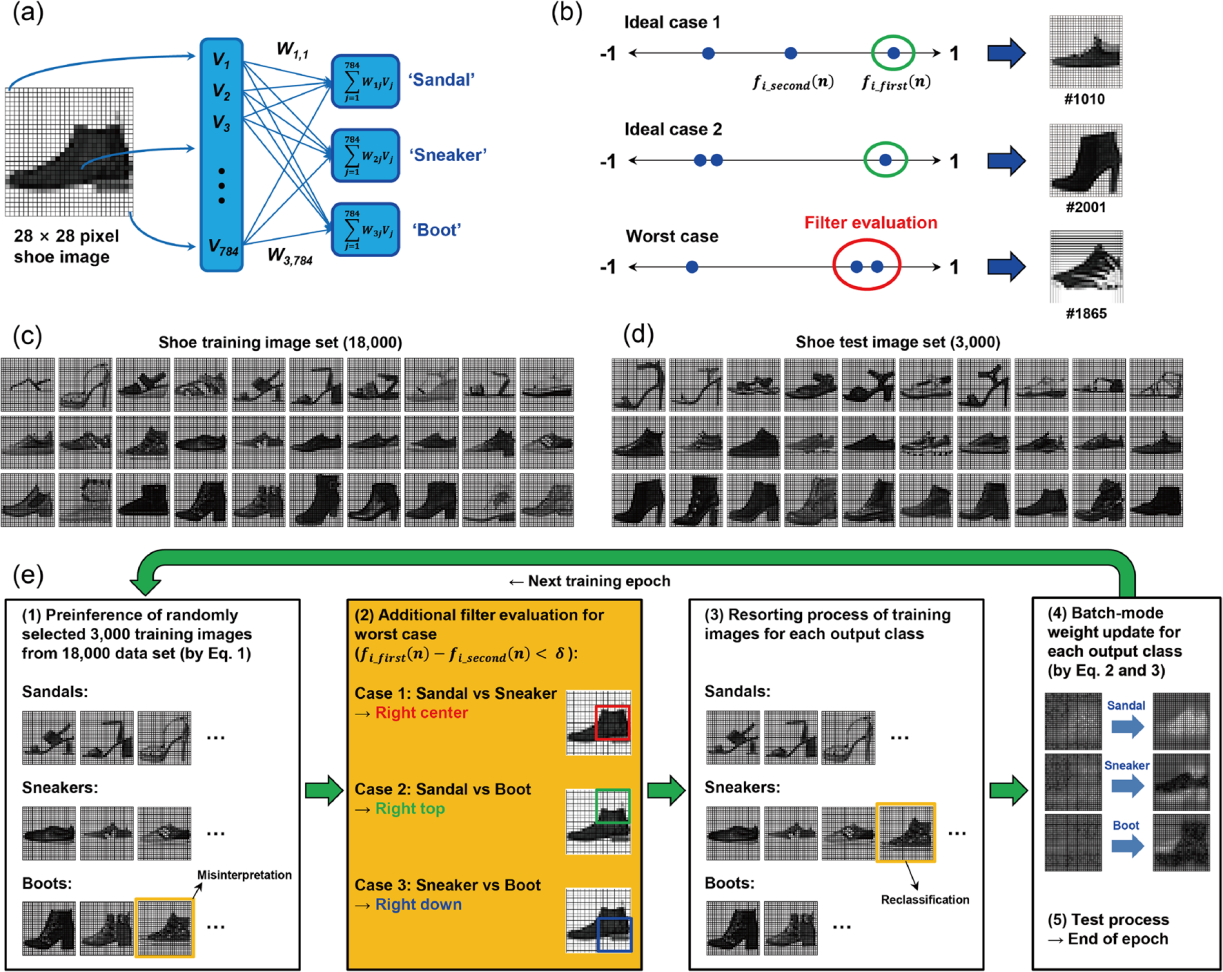
(**a**) Constituents of FCSN for the 28 × 28 shoe image inference process. Each input pixel is linked to the output neurons consisting of ‘Sandal’, ‘Sneaker’ and ‘Boot’ with a synaptic weight. (**b**) Configuration of the activation function showing 3 output results for each input image (between − 1 and 1) for the ideal cases (upper and middle line) and worst case (lower line). (**c**) 18,000 training images and (**d**) 3,000 test images for the shoe data set. (**e**) Flow chart of one epoch of the training/test algorithm, where the yellow box shows that the filter evaluation is only executed if the difference between the first and second activation function values is smaller than the predetermined δ-value.

The main task is shoe classification solved by vector–matrix calculation of the analogue state level in the FCSN system, as represented in Fig. 1a. Each shoe image consists of 784 (28 × 28) pixels with 256 levels of grayscale^32^, which are transformed to the magnitude of the input bias between − 0.5 and 0.5 V. The input neuron is fully connected to a 3-class output neuron, in terms of ‘Sandal’, ‘Sneaker’ and ‘Boot’, with individual connecting strengths as the synaptic weight. The conventional decision method is based on a sequence of vector–matrix multiplication results between the input bias and the selected synaptic weight that is transformed to non-dimensional parameters via the tangent hyperbolic function to limit the output signal in the range from − 1 to 1 as follows^31,33,34^:

1 $$f_{{\text{i}}} \left( {\text{n}} \right) \, = {\text{ tanh }}(\beta \sum {\text{w}}_{{{\text{ij}}}} {\text{V}}_{{\text{j}}} )$$

where β is the coefficient used to determine the slope of the activation function, w_ij_ is the weight matrix element, and V_j_ is the pixel intensity of input image (j = 1–784). From this decision rule, a number of image classifications are quickly available in the FCSN system, but an intrinsic limitation exists because the overall arrangement of the output results is not of concern in this step. Indeed, the activation function values of the undesignated output neurons are quite important because they also affect the weight increment process in a negative direction and modify the synaptic weight matrix according to the classification results at each training epoch^30^. If the second highest output neuron is the targeted answer and first output neuron is the inferred answer in confusable classification, this situation inevitably causes a considerably incorrect weight update of the correct image in the backpropagation learning rule^30,31^. The specific example is represented in Fig. 1b with the configuration of activation function values for a few shoe images obtained by simulation in a FCSN system. For the ideal case 1, the output result is well distributed in an overall range, and the first highest value clearly corresponds to ‘Sneaker’ (1010th image: − 0.88165 for Sandal, − 0.57557 for Boot, and − 0.38362 for Sneaker). Similarly, the decision for the ideal case 2 is also obvious (2001th image: − 0.87317 for Sandal, − 0.81738 for Sneaker and 0.2716 for Boot) because the highest activation function value for the output neuron of ‘Boot’ is located far away from the other values. However, in the worst case (1865th image: − 0.9034 for Sandal, − 0.50689 for Sneaker and − 0.46234 for Boot), the first and second highest activation function values are so similar that classification is notably difficult between ‘Sneaker’ and ‘Boot’. To overcome this confusion, we established an additional auxiliary network that links a portion of the image pixel as an input bias to the competitive output neurons, and it works only in a particular case when the differences of the first and second highest activation function values are too small to distinguish, i.e., less than the designated limit value.

### Run-off election-based simulation algorithm

For implementation of the run-off election decision method in the FCSN system, first, we preliminarily classified the 3,000 training shoe images that were randomly selected from the 18,000-image data set (Fig. 1c) using the conventional decision rule. Because random selection of the training image was different in each training epoch, the reliability of simulation can be enhanced with reflecting complex data variability^35^. In this work, we applied an additional evaluation step to perform a resorting process for the confusable image that shows a smaller difference between the first and second activation function values than the predetermined δ-value by filter evaluation according to the case of competitive classes. For example, if there was confusion between ‘Sandal’ and ‘Sneaker’, resorting was performed by vector–matrix multiplication between the input bias in the 196 pixels of the right center (RC) and a 2-class output neuron, similarly for the case of ‘Sandal’ and ‘Boot’ in the right top (RT) and ‘Sneaker’ and ‘Boot’ in the right down (RD). The classified results, including the results of filter evaluation, were transferred to calculation of the weight increment for each synaptic network using the conventional batch-mode delta rule weight updating process, as shown in the following Eqs. ^33,34^:

2 $$\Delta_{{{\text{ij}}}} \left( {\text{n}} \right) \, = \, [f_{{\text{i}}}^{{({\text{g}})}} \left( {\text{n}} \right){-}f_{{\text{i}}} \left( {\text{n}} \right)]{\text{ d}}f/{\text{dI V}}_{{\text{j}}} \left( {\text{n}} \right)$$

3 $$\Delta {\text{w}}_{{{\text{ij}}}} = \alpha \sum \Delta_{{{\text{ij}}}} \left( {\text{n}} \right)$$

where *f*_i_^(g)^(n) is the target value of the *i*-th output neuron for the *n*-th input image, ‘I’ means ∑w_ij_V_j_ (Eq. 1), ∆w_ij_ is the updated weight value for *j*-th pixel of *i*-th output, and α is the device parameter determined by the conductance change at the given conductance value which is obtained by fitting the long-term potentiation/depression (LTP/LTD) curves of the synaptic device because each synaptic weight element corresponds to conductance in synaptic device cell (Supplementary Table 1). Among the various pulse configurations used to obtain the LTP/LTD curves, we focused on the specified condition at a pulse magnitude of − 1.65 V and width of 100 ms. The simulation results for different LTP/LTD curves are presented in Supplementary Fig. 1. The weight updating processes for the 3 types of auxiliary network were generally identical to that of the main network, but they differed in the number of pixels (196) and output (2) elements. Subsequently, 3,000 test images (Fig. 1d) that were not used in the training process were evaluated by ones based on the updated synaptic weight matrix after each training epoch. Figure 1e summarizes one epoch of the run-off election decision algorithm using filter evaluation (yellow box) that implements the resorting process via the 2 × 196 sized auxiliary network according to the competitive classes.

## Results and discussion

### Effect of filter evaluation for the inference process

**Figure 2:**
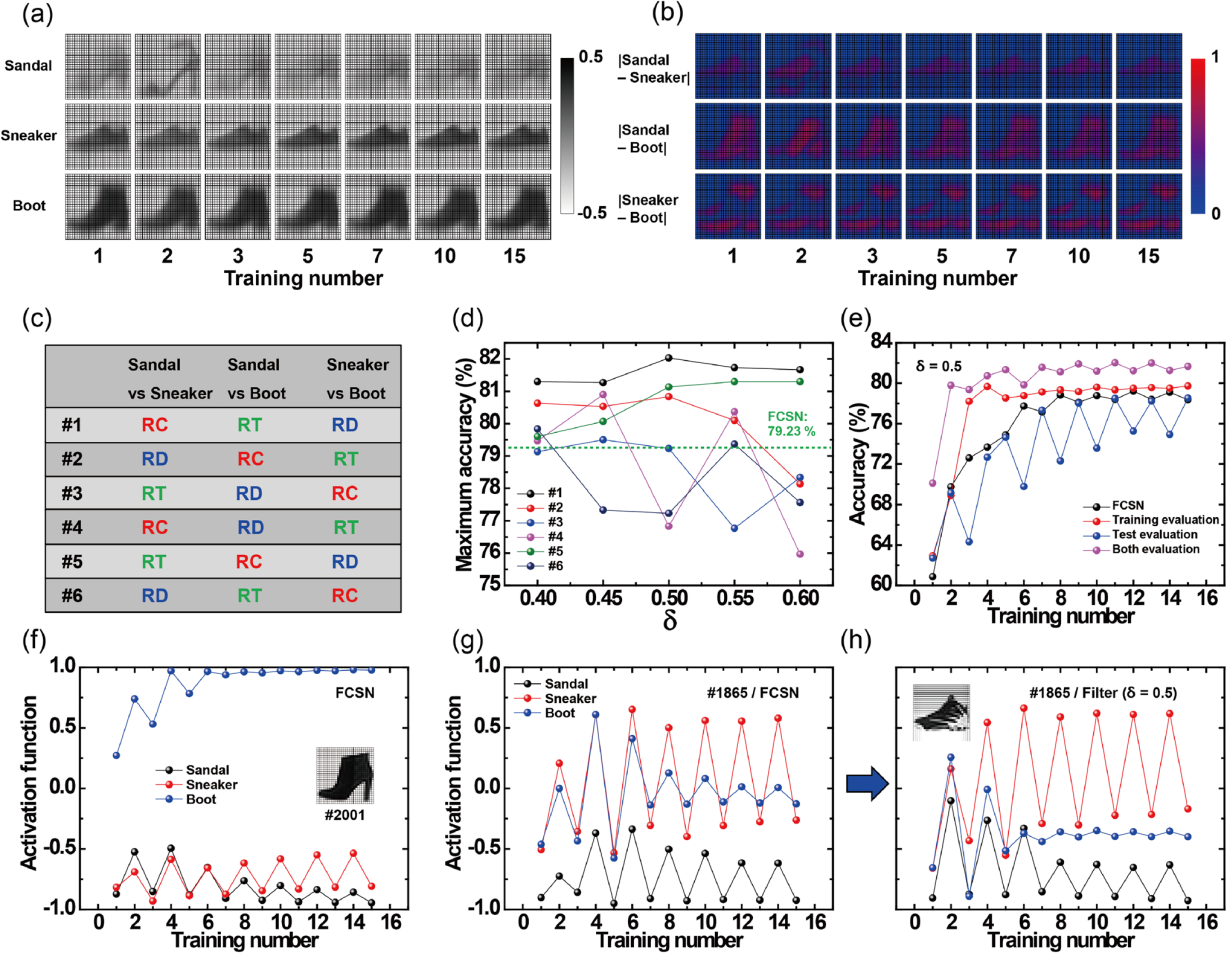
(**a**) Average magnitude of the classified training images for each output class at each training epoch in FCSN and (**b**) absolute difference between the two classes, where the red pixel indicates the more distinguishable region and the blue pixel indicates a similar region. (**c**) Arrangement of the 3-filter location along the competitive output classes. (**d**) The maximum inference accuracy in 15 training numbers depends on the filter arrangement in (**c**), where the #1 case (black line) showed the best improvement at a δ-value of 0.5 compared with the result in the FCSN without filter evaluation (green dash line). (**e**) Evolution of inference accuracy during 15 training numbers for the FCSN (black line) and with filter evaluation only in the training process (red line), only in the test process (blue line), and in both the training/test processes (magenta line). Evolution of activation function values during 15 training numbers for test image (**f**) #2001 and (**g**) #1865 in the FCSN. (**h**) Enhanced separation of activation function values for test image #1865 with filter evaluation at a δ-value of 0.5.

Figure 2a shows the overlapped intensity of the classified training images in FCSN for each output class during the training process. Based on this common feature, we obtained the absolute difference contour map between the two output classes (nC_2_ cases for n of total output) as shown in Fig. 2b, where the red pixel region shows the more different portion to be selected as filter location while the blue pixel indicates the similar portion. For competition between Sandal and Sneaker, the RC portion (rear end of shoes) showed the most distinguishable intensity, i.e., the appropriate location for the filter evaluation. On the same principle, the RT (ankle portion) and RD (heel portion) region were chosen as an auxiliary filter location for Sandal-Boot and Sneaker-Boot evaluation, respectively. To confirm the availability of this selection, we investigated the filter-evaluated simulation for other configurations of filter location in the RC, RT, and RD regions, as shown in Fig. 2c. The abovementioned filter arrangement is relevant to case #1, which presented the highest accuracy improvement of over ~ 82.03% (black line in Fig. 2d) at a δ-value of 0.5 compared with the original accuracy value of ~ 79.23% in the FCSN (green dash line). The inference results showed variability along the configuration of filter location (e.g., case #4 exhibited a considerable decrease in accuracy), which implied the importance of the proper assignment of filter location along the competitive classes. The statistical results for the filter-evaluated simulation are presented in Supplementary Fig. 2.

Figure 2e shows comparison of the inference results at different filter application ranges for a δ-value of 0.5. The result for the FCSN is plotted using a black line, which corresponds to a convergence of ~ 79.23% (green dash line in Fig. 2d). When the filter evaluation was partially applied in either the training (red line) or the test (blue line) step, no significant improvement was observed because application in the test process only could not derive the weight matrix change during the training epoch, and only the training process could not utilize the filter decision method. The result of filter evaluation in both the training/test process is represented by a magenta line, which presented remarkably enhanced performance with a maximum accuracy of ~ 82.03%. To assess the propagation of the activation function values in the effect of filter evaluation, we analyzed the evolution of the activation function for the test images relevant to the ideal and worst cases (2001th and 1865th images in Fig. 1b) during the training epoch. Figure 2f shows the result for the ideal case classified as ‘Boot’ for the 2001th test image in the FCSN. The activation function value for the output neuron of ‘Boot’ (blue line) shows clear superiority compared with the other output results from the beginning, and its separation was also gradually increased. In this ideal case, filter evaluation is not required because of a wide gap between the first and second highest output results. However, when the first and second output results were sufficiently similar, as in the 1865th image, the classification task was notably difficult because of a continuous incorrect weight update from misjudgment of these images. Indeed, for this case, the confusion of the output result between ‘Sneaker’ and ‘Boot’ persisted during the entire training epoch in the FCSN (Fig. 2g). In such a worst case, filter evaluation can be usefully applied to separate the competitive output class. Because the output result for the 1865th image shows confusion between ‘Sneaker’ and ‘Boot’, the filter evaluation focused on the RD filter (#1 condition in Fig. 2c) with a δ-value of 0.5. The inference result for the filter evaluation is presented in Fig. 2h, where clear separation of competitive output classes can be observed as the training number increases (red and blue line), producing a reliable decision for ‘Sneaker’ with the aid of a modified weight matrix update from the resorting process of confusable images.

**Figure 3:**
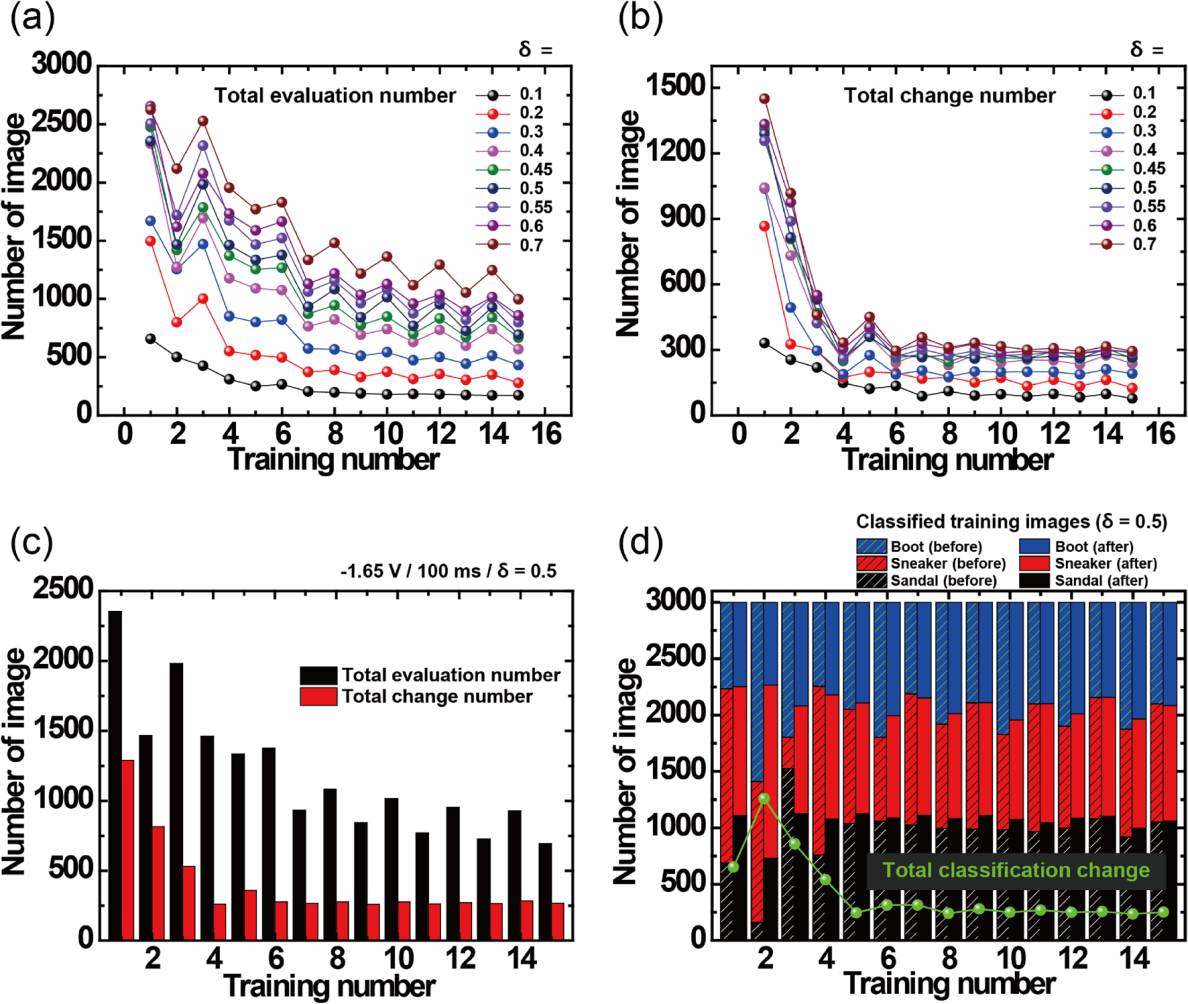
Number of (**a**) total evaluations and (**b**) changes for test images during 15 training numbers with δ-value variation from 0.1 to 0.7. (**c**) Total number of filter-evaluated test images (black bar) and classification changes (red bar) during 15 training numbers at a δ-value of 0.5. (**d**) Classified numbers of training images before (solidus bar) and after (filled bar) filter evaluation during 15 training numbers at a δ-value of 0.5, where the green line represents the number of total classification changes at each epoch.

In the run-off based decision method, the number of evaluation runs is entirely dependent on the conditional constraint of the δ-value because it eventually determines whether the filter evaluation is used at a given difference value between the first and second activation functions. Consequently, it is evident that the number of evaluations could be increased at a larger δ-value. However, a high evaluation number does not always mean greater enhancement of inference accuracy because excessive evaluation definitely contains images that do not need to be evaluated. Figure 3a,b shows the number of total evaluations and changes for the test images during the filter-evaluated training process with δ-value variation. Here, the decreasing tendency of the evaluation number during training process is particular point of conditionally working run-off election-based decision method unlike the consistent filter operation in conventional convolution network system^15,36^. Although both graphs showed an increasing tendency for higher δ-value, the variation in the total change number was not significant compared with the remarkable increase in evaluation number, which indicates that an excessively high δ-value induced unnecessary evaluation and misjudgment. Actually, the simulation results for filter evaluation presented an optimized δ-value near 0.5 to obtain the maximum inference accuracy (Supplementary Fig. 1c). Figure 3c summarizes the result for a δ-value of 0.5, where it can be easily observed that the total change number (red bar) is relatively invariant to the total evaluation number (black bar). This result indicates that the application of filter evaluation should be confined only to confusable images. The classification changes for the training image are presented in Fig. 3d, where the left solidus and right filled bars at each training number indicate the distribution of classified images before and after filter evaluation at a δ-value of 0.5, respectively. In this work, because 3000 training images were randomly selected in a set of 18,000 images at each training number, the training images differed for each epoch, reflecting complex data variability for the simulation. The number of total classification changes are shown by the green line, which indicates that most of the changes were concentrated in the initial training epoch before stabilization of the weight matrix. These results are also related to the notable increase in inference accuracy with filter evaluation, especially at low training numbers (Fig. 2e). Because the sufficiently updated weight matrix become insensitive to additional training steps after a repetitive training process, the correct formation of the weight matrix configuration in the initial step is particularly important^33^. The simulation results for the sigmoidal activation function and without the device parameters are presented in Supplementary Fig. 3.

### Synaptic weight updating procedure in the main and auxiliary network

**Figure 4:**
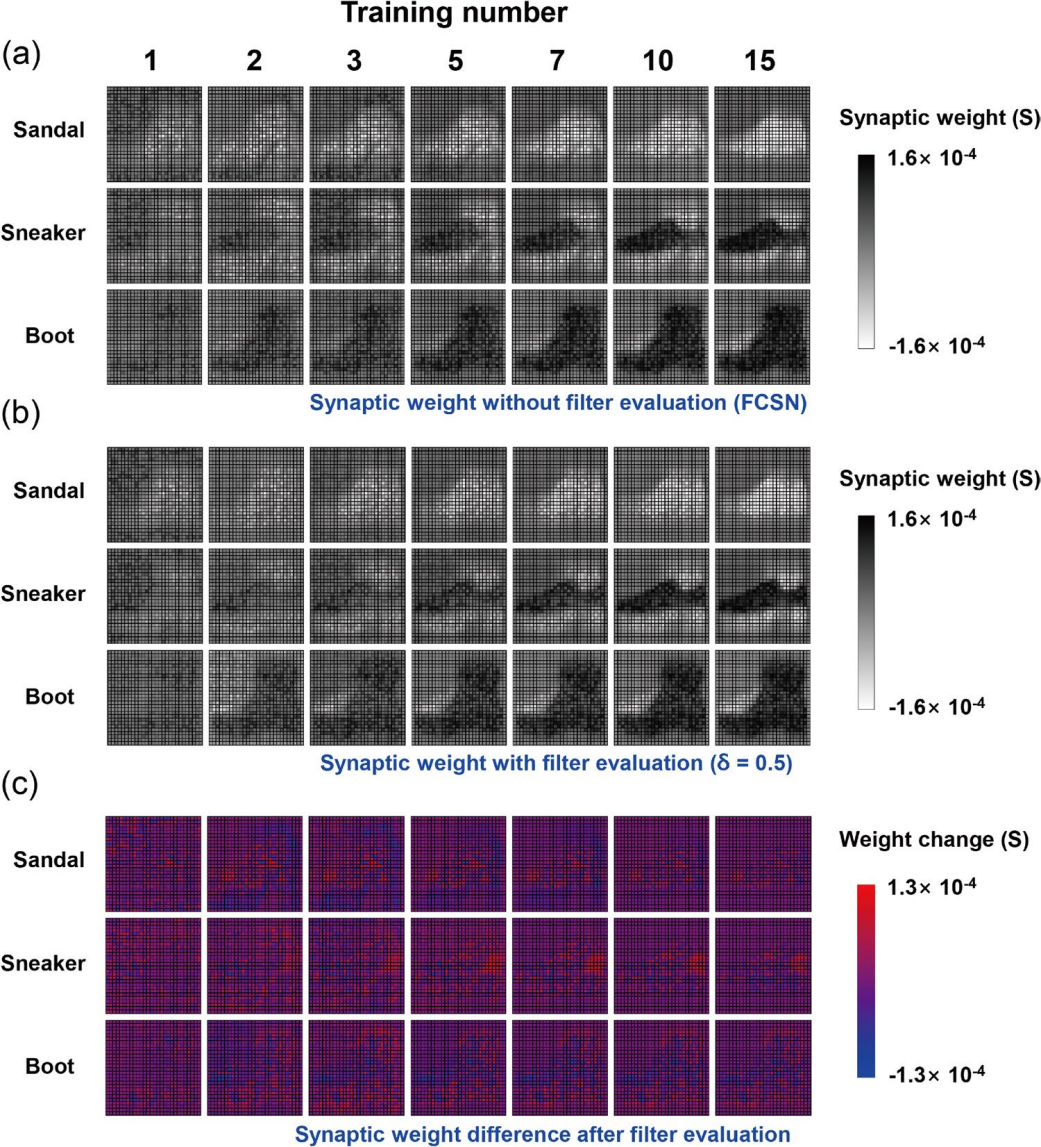
Reshaped 28 × 28 contour images of the synaptic weight for each output class during 15 training numbers (**a**) without and (**b**) with filter evaluation at a δ-value of 0.5 and (**c**) their difference.

**Figure 5:**
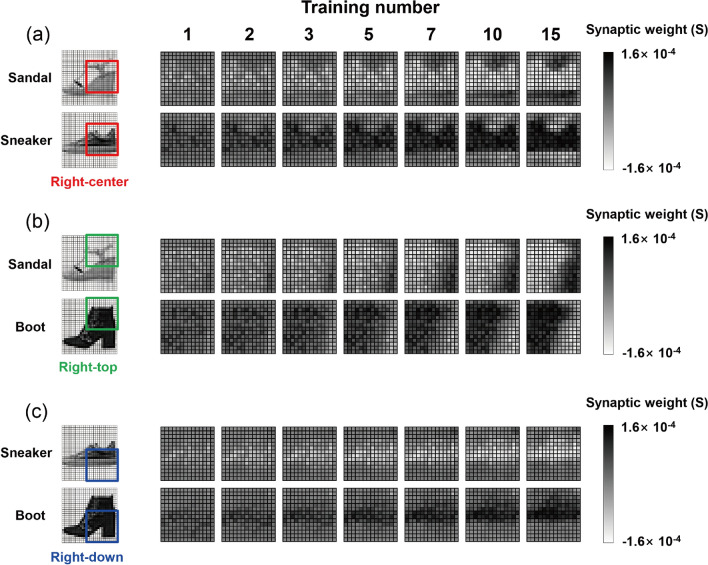
Reshaped 14 × 14 contour images of the synaptic weight of each filter with filter evaluation at a δ-value of 0.5 during 15 training numbers for (**a**) right-center filter to divide ‘Sandal’ and ‘Sneaker’, (**b**) right-top filter to divide ‘Sandal’ and ‘Boot’, and (**c**) right-down filter to divide ‘Sneaker’ and ‘Boot’.

Figure 4a presents the variation of the synaptic weight for each output neuron as the training numbers increase in the FCSN system. Because we used a pair of relevant matrices at each output neuron to link the conductance to the synaptic weight, it was confined in a range of ± (G_max_ − G_min_)^33,34^. As the training process continued, the contour map of the weight matrix gradually converged to the shape of the relevant output image because the commonly positioned pixels at each output result received positive weight updates, whereas those of the emptied pixels received negative weight updates according to the learning rule^30,31^. For example, the positioned pixel of ‘Boot’ enhanced the synaptic weight at the relevant output neuron (black pixel region in the Boot weight matrix) with the training process, but it worked in the negative direction at other output neurons (white pixel region in the Sandal and Sneaker output neurons). Additionally, when the filter evaluation was performed with the shoe resorting process, the propagation of the weight update showed some difference compared with the result in the FCSN. Figure 4b shows the synaptic weight with filter evaluation at a δ-value of 0.5, and the difference value is replotted in Fig. 4c, where a certain portion of the weight element (for example, rear end of Sneaker) was remarkably changed by the effect of classification change in the training process. This detail can also be observed in the synaptic weight of the auxiliary network, as shown in the RC filter (determination of Sandal or Sneaker) in Fig. 5a, where the synaptic weight of the rear end for ‘Sneaker’ was intensively enhanced via filter evaluation. An interesting point is that the positive weight update quantity in the particular neuron directly affected the other neuron as a result of the negative weight update because of the 2-class filter evaluation in the auxiliary network. In this manner, the complementary weight update process was achieved in RT (Fig. 5b), and the RD (Fig. 5c) filter correctly guided the direction of the synaptic weight update in the main network system. Because the classification change in the training step ultimately results in a change of synaptic weight propagation in all network architectures, a possible interpretation is that the update of the auxiliary network is related to that of the main network system despite its independent synaptic connection, and it is sufficient to infer the main synaptic weight matrix through configuration of the auxiliary network. The synaptic weight propagation of filter-evaluated simulation without device parameters is given in Supplementary Fig. 4 (main network) and Fig. 5 (auxiliary network). In addition, non-square filter application for the generalized data set is presented in Supplementary Fig. 6. Based on the common feature map of the classified output classes, it was enabled to constitute filter shape and location to efficiently separate the competitive output classes, resulting in dramatic enhancement of inference performance of the complex network system.

## Conclusion

In summary, we designed a conditionally working auxiliary network to precisely process confusable image classification in the ANN system. By optimizing the constraint of the δ-value and spatial position of each auxiliary network, we could control the quantity of the filter evaluation process and enhance the inference accuracy for the shoe image data set while avoiding misinterpretation caused by confusable configuration of the activation function values. The run-off election-based decision method offers a novel classification rule that can improve the training and inference performance without interruption of the original decision rule based on vector–matrix multiplication, and provides the availability for generalized data set with the selection of filter shape and location based on the common feature of the classified output classes. This method shows the ability to induce a modified synaptic weight update at each node in an array network with the independent auxiliary network. Because the decision step for the output neuron is essential for all types of ANN systems, we believe that this run-off election-based filter evaluation method can be used in a more complex network architecture with superior applicability and effectiveness.

## Supplementary information

Supplementary Information.
