# Supplementary material for: Run-off election-based decision method for the training and inference process in an artificial neural network
Source: Sci Rep. 2021 Jan 13;11:895. doi: 10.1038/s41598-020-79452-2 (PMC7806707; doi:10.1038/s41598-020-79452-2)
Supplement: Supplementary file 1 — Supplementary Information. [file 41598_2020_79452_MOESM1_ESM.doc]

**Supplementary Information**

**Run-off election-based decision method for the training and inference process in an artificial neural network**

Jingon Jang*, Seonghoon Jang, Sanghyeon Choi & Gunuk Wang*

KU-KIST Graduate School of Converging Science and Technology, Korea University, 145, Anam-ro, Seongbuk-gu, Seoul 02841, Republic of Korea

*E-mail:jangjg@korea.ac.kr; [gunukwang@korea.ac.kr](mailto:gunukwang@korea.ac.kr)

1. Simulation results for different pulse configurations of the synaptic devices

The analogue switching curves used in the simulation process were obtained in electrical measurement of conventional titanium oxide resistive memory devices. As shown in Fig. S1a, the analogue conductance state was measured as the postsynaptic current (PSC) in the grounded bottom electrode at a read voltage of -0.5 V while the presynaptic pulse was applied on the top electrode. In this work, the vertical layer structured synaptic memory device was fabricated on a bare glass substrate using thermal evaporation (bottom and top Al electrode of ~30 nm) and a RF sputtering process (active titanium oxide layer of ~20 nm), as previously reported^S1^. According to the presynaptic pulse configuration (pulse amplitude/width = -1.55 V/100 ms for the black line, -1.65 V/100 ms for the red line, and -1.6 V/150 ms for the blue line at a fixed depression amplitude of 1.4 V and pulse period of 800 ms), the analogue conductance switching curves showed different long-term potentiation/depression (LTP/LTD) plots and fitting coefficients of the conventional formulae as shown in the following equations^S2^:

ΔG_LTP_ (G) = *a_p_* + *b_p_* exp [–*c_p_**(G–G_min_)/(G_max_–G_min_)] (1)

ΔG_LTD_ (G) = *a_d_* + *b_d_* exp [–*c_d_**(G_max_–G)/(G_max_–G_min_)] (2)

Based on these fitting coefficients, the degree of conductance change can be determined at all conductance state to reflect the synaptic device parameter on synaptic weight update in the training process. The calculated fitting coefficients in different pulse configurations are arranged in Table S1, and the simulation results for each LTP/LTD curve in the fully connected single layer network (FCSN) are presented in Fig. S1b. Unlike the difference in the fitting coefficient, the inference accuracy showed a similar value near ~79.23 % regardless of the pulse configurations because the LTP/LTD curve only affects the training rate of the weight increment/decrement at a fixed delta-rule weight change as ∆_ij_ (Eq. 2 in main text), which could converge in repetitive training steps. However, for the simulation with filter evaluation, classification change at each training epoch resulted in a ∆_ij_ change. Therefore, a remarkable change in inference accuracy was observed in the filter evaluation for all pulse configurations, as shown in Fig. S1c, where the optimized δ-value near 0.5 to extract maximum accuracy was recovered, as shown in Fig. 2d in main text. The evolution of inference accuracy during the training epoch for all pulse configurations is presented in Fig. S1d-f. It can be clearly observed that the propagation of the accuracy plot near δ-values of 0.4-0.5 is located far away from that of the other δ-values during the entire training range in all pulse configurations.


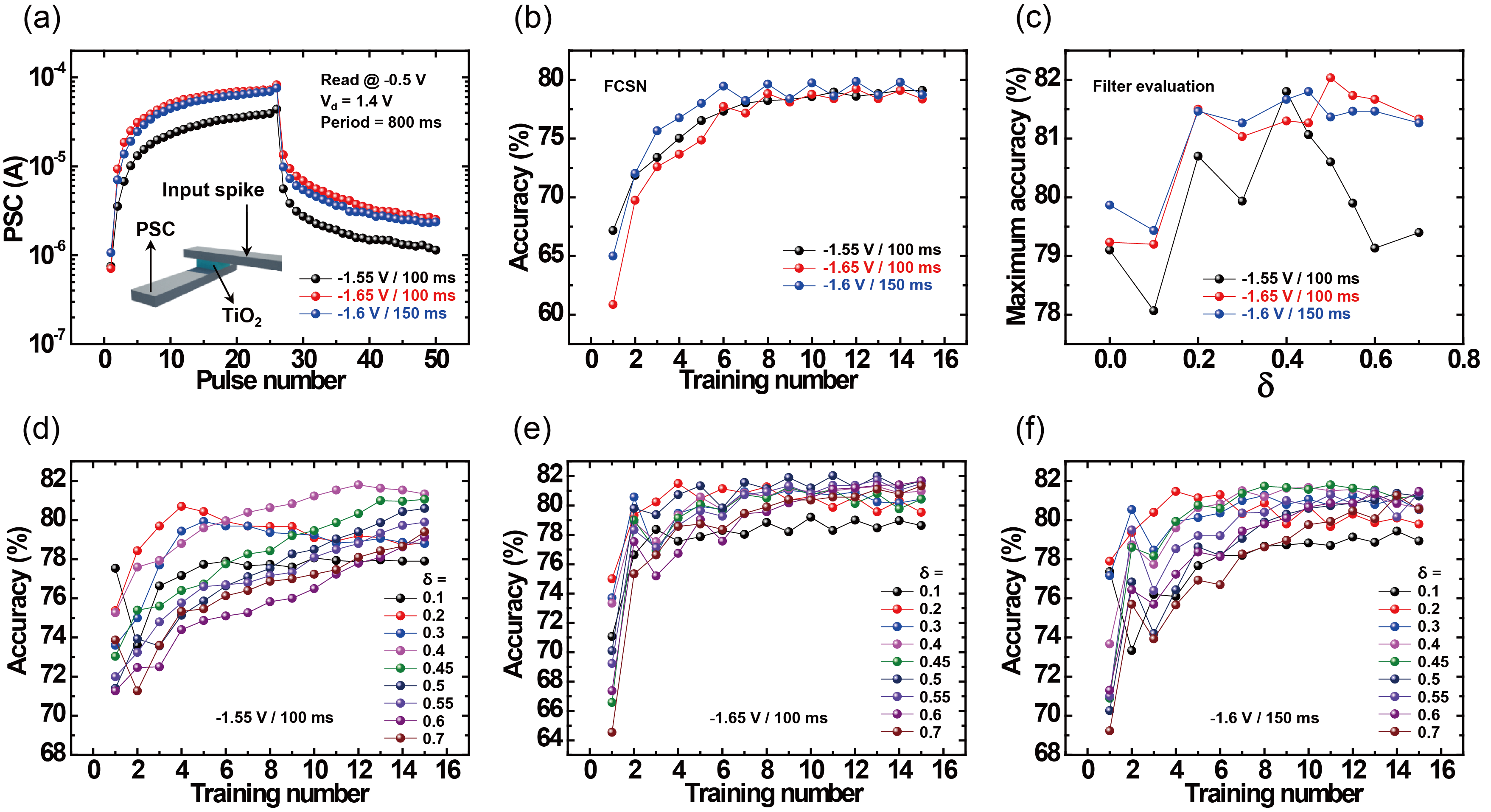


**Figure S1.** (a) LTP/LTD characteristics of the TiO_2_ synaptic device for different potentiation pulse configurations (amplitude/width) at a pulse period of 800 ms. Inset shows the vertical layer structure of the device presenting the direction of the input spike and PSC at each electrode line. (b) Evolution of inference accuracy during 15 training numbers for FCSN at different input pulse configurations (amplitude/width) at -1.55 V/100 ms (black line), -1.65 V/100 ms (red line), and -1.6 V/150 ms (blue line). (c) Maximum inference accuracy in 15 training numbers with δ-value variation from the FCSN of δ = 0 to 0.7 at different input pulse configurations. The evolution of inference accuracy during 15 training numbers with δ-value variation from 0.1 to 0.7 is presented for the different pulse configuration at (d) -1.55 V/100 ms, (e) -1.65 V/100 ms, and (f) -1.6 V/150 ms.

**
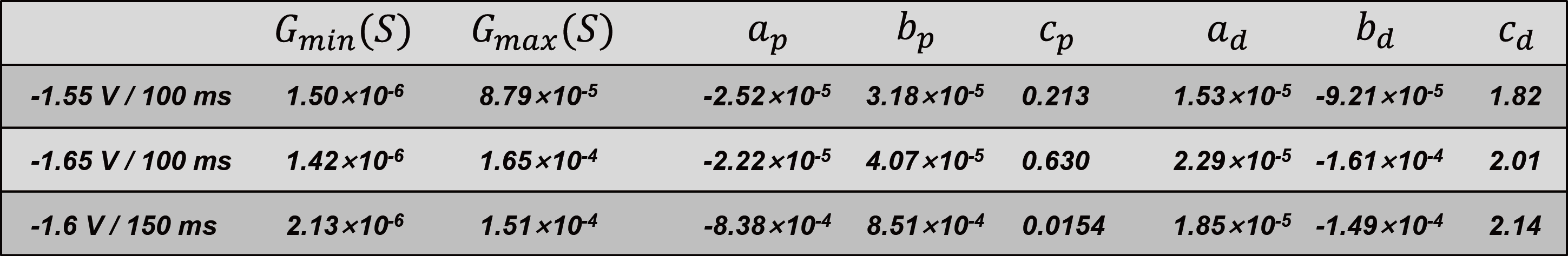
**

**Table S1.** Fitting parameter used to decide the conductance increment/decrement depending on the conductance value obtained by the LTP/LTD curves at different pulse configurations.

**2. Statistical simulation results and modification of the simulation function**

Because selection of the training image differs at each epoch to reflect the complex data variability, deviation of the output results could occur in both the FCSN and filter evaluation at each implementation. However, the training process is based on the calculation from common features in the classified data set, and the repetitive training steps can ensure that the comprehensive output results can converge^S3^. Figure S2a shows the evolution of inference accuracy in the FCSN with 5 identical implementations of the simulation. Despite the slight difference in accuracy results at low training number, the statistics showed a significantly low deviation of accuracy of 0.186 % in the 15th epoch. Additionally, in the filter evaluation (0.5 of δ-value), the deviation value showed a low value of 0.166 %, as shown in Fig. S2b, indicating the reliability of the simulation process. Figure S2c shows the cost function of training process which statistically summarizes the average of error value for each activation function of 3,000 test images. As training process are implemented, the designated activation function approach to ‘1’ and that of undesignated case approach to ‘-1’, resulting in continual reduction of the cost function. The total error distribution of several training epoch (2, 4, 10, and 15) is represented in Fig. S2d-e for FCSN and filter evaluation (0.5 of δ-value), respectively.


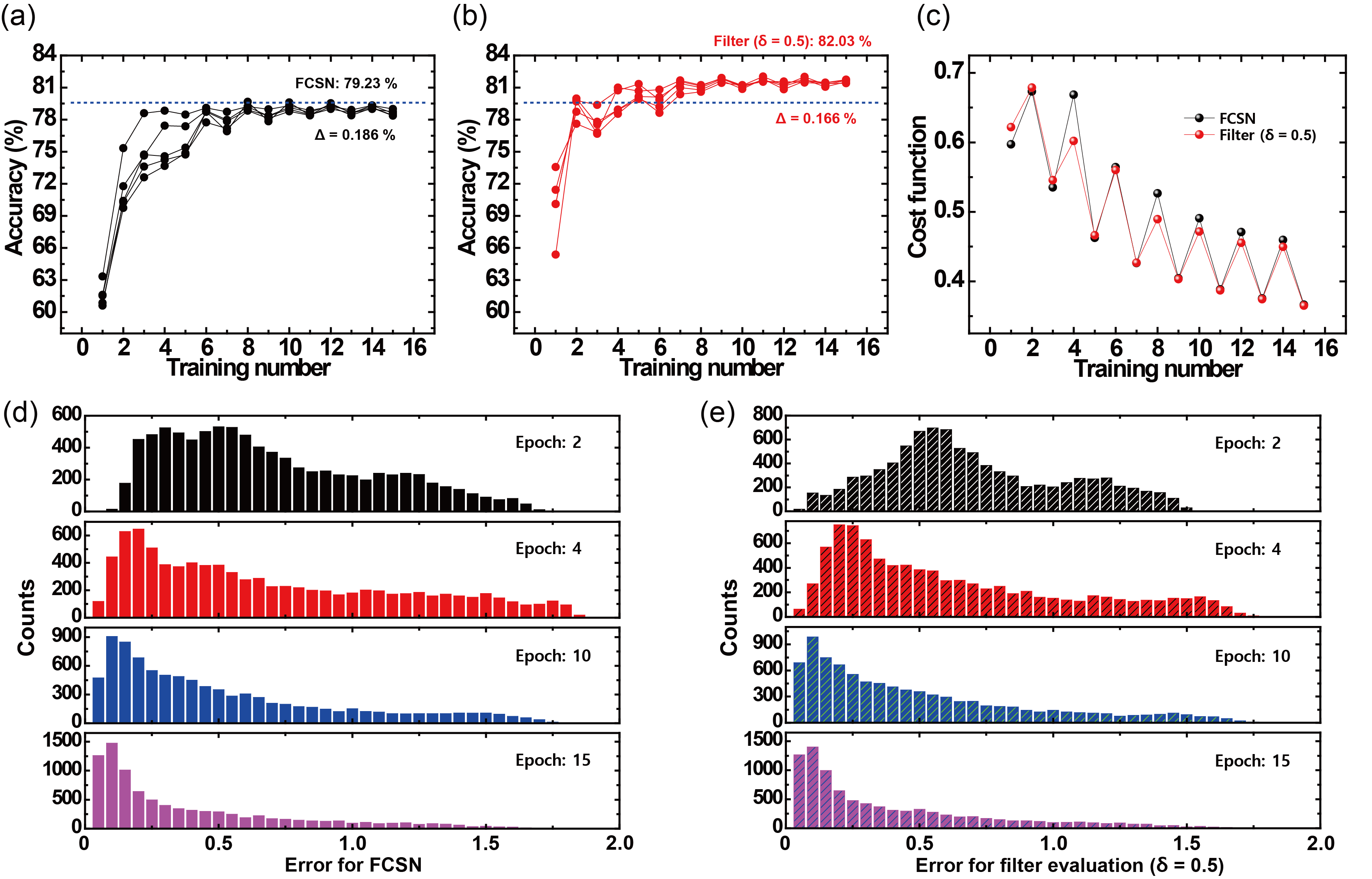


**Figure S2.** Statistical simulation results for (a) FCSN and (b) filter evaluation (δ-value of 0.5) as the evolution of inference accuracy at the training epoch where the identical simulation algorithm was executed 5 times to obtain the standard deviation (Δ) of the accuracy. (c) The cost function for training process of FCSN (black line) and filter evaluation (δ-value of 0.5, red line). Histogram for 9,000 number of error values for several training epoch of (d) FCSN and (e) filter evaluation (δ-value of 0.5).

For the FCSN and filter-evaluated simulation, the activation function used to determine the magnitude of output signal was selected as a tangent hyperbolic function (Eq. 1 in main text) to monotonously transform the input signal to a range between -1 to 1. When we changed the activation function to sigmoidal, i.e., *f*_i_(n) = 1/(1+exp[–β∑w_ij_V_j_]), the simulation process was identical, but the change in the δ-value was inevitable due to the reduced output range between 0 to 1. Fig. S3a shows the evolution of the inference accuracy of the sigmoidal function-based FCSN (black line) and filter evaluation (δ-value of 0.05, red line), resulting in obvious accuracy enhancement with filter evaluation in all training epochs. The result of performance variation according to different δ-values is presented in Fig. S3b, where the maximum accuracy is located at a δ-value of 0.05, and the results for δ-values over 0.06 showed accuracy similar to the original performance in the FCSN because such a level definitely contains images that do not need to be evaluated (Fig. 3a-b, main text). Figure S3c shows the number of evaluated test images in the sigmoidal function-based filter evaluation, which indicates similar decreasing and saturation behavior as shown in Fig. 3c of the main text. For the equation of the batch-mode delta rule weight updating process (Eq. 3 in main text), if α is replaced by a numerical constant (10^7^) of the learning rate, it is possible to extract the results of the numerical simulation excluding the synaptic device parameter of LTP/LTD curve, that is, ∆w_ij_ can be proportional to ∑∆_ij_(n). The simulation principle is identical to that of the device parameter for the FCSN and filter evaluation in constraint of the δ-value, resulting in a similar inference accuracy (Fig. S3d), maximum accuracy plot (Fig. S3e), and number of evaluated images (Fig. S3f). Consequently, the simulation procedures with filter evaluation were not significantly affected by a case of activation functions or even in the device parameters, indicating the potential availability of filter-evaluated decision rule for the generalized training and inference process with various network parameters.


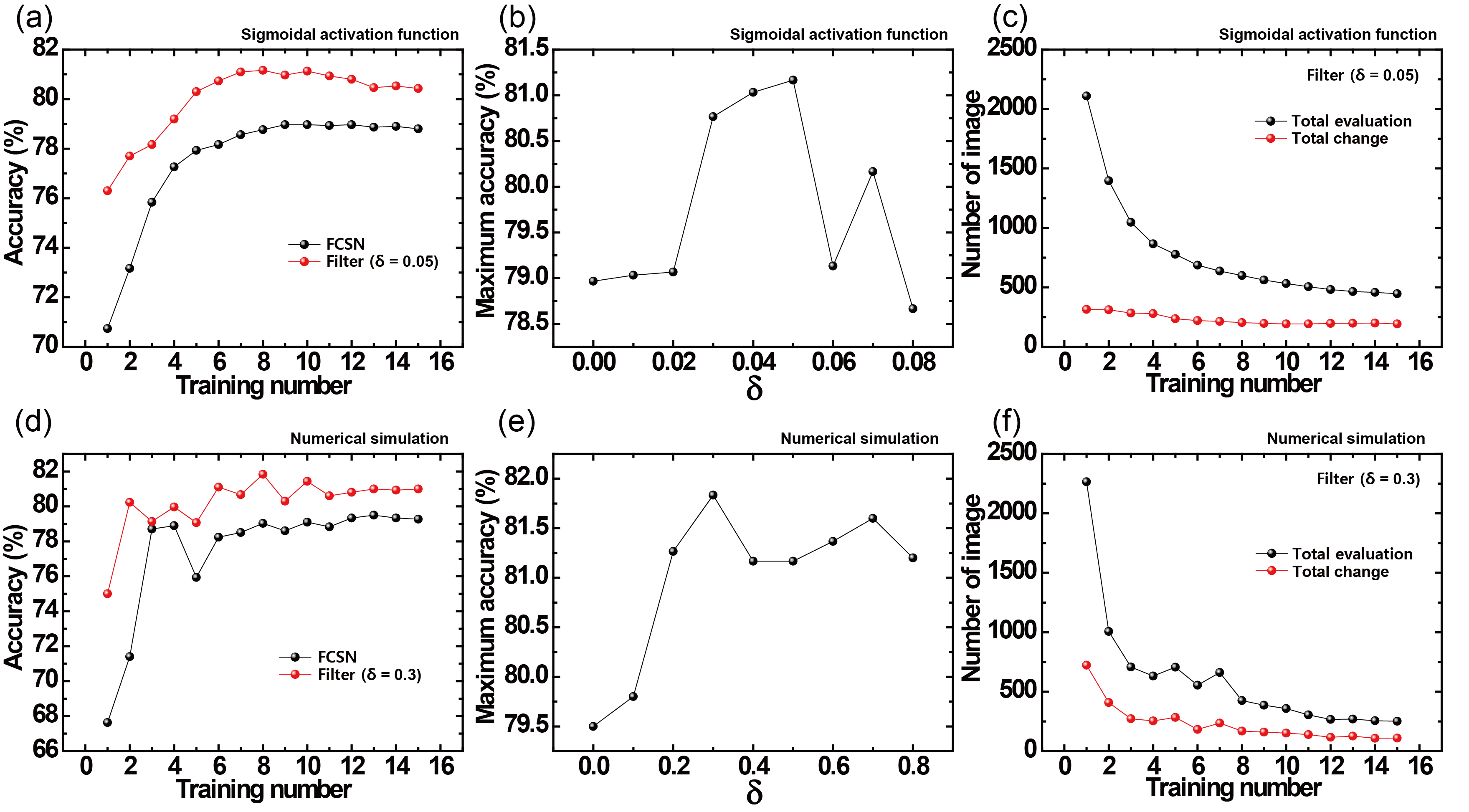


**Figure S3.** (a) Evolution of inference accuracy at the training epoch, (b) maximum accuracy for different δ-values, and (c) number of filter-evaluated test images for the simulation using the sigmoidal activation function. (d-f) Results for the numerical simulation without the synaptic device LTP/LTD characteristics.


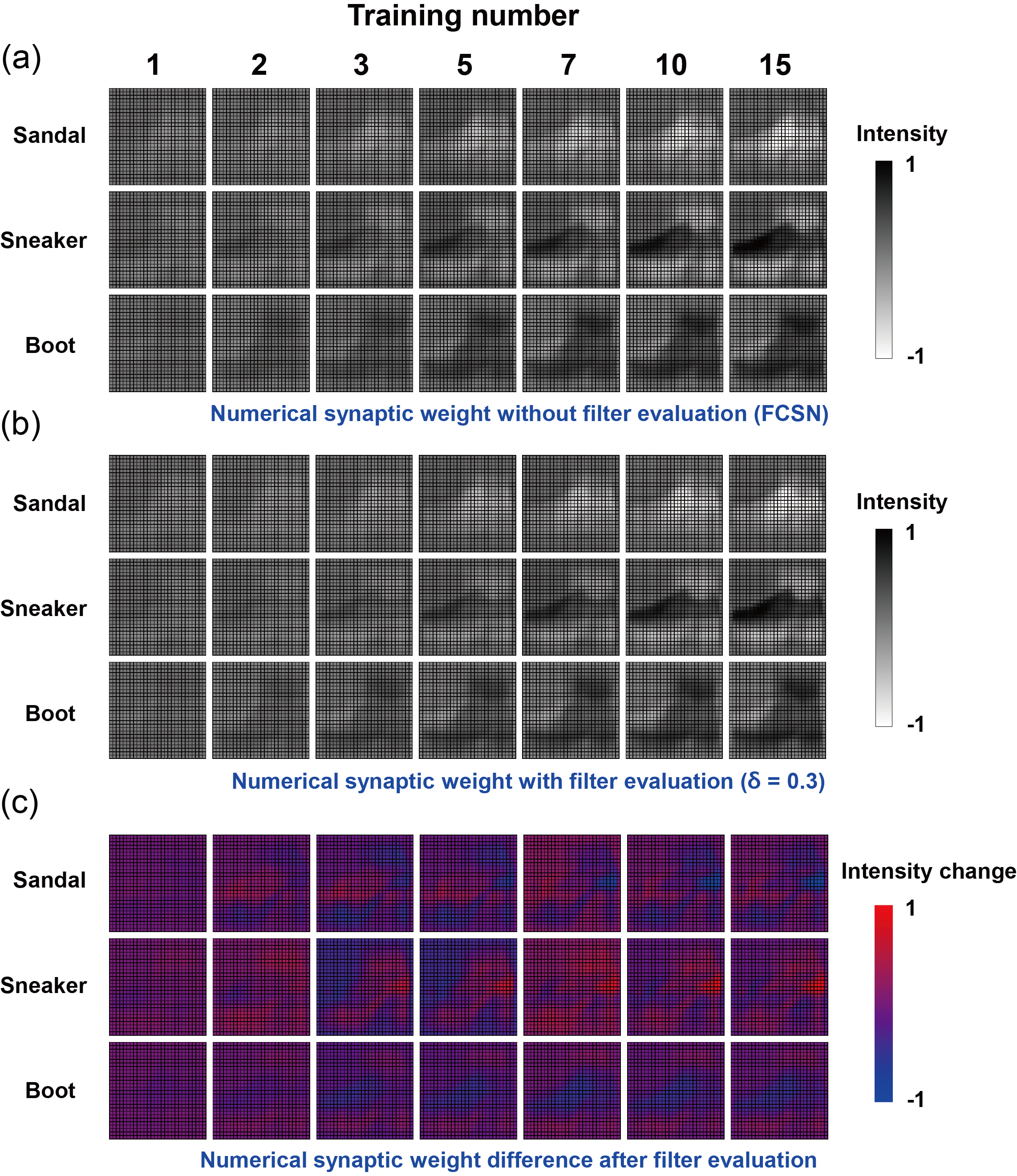


**Figure S4.** Reshaped 28 × 28 contour images of the synaptic weight for numerical simulation (a) without and (b) with filter evaluation at a δ-value of 0.3 and (c) their difference.

**
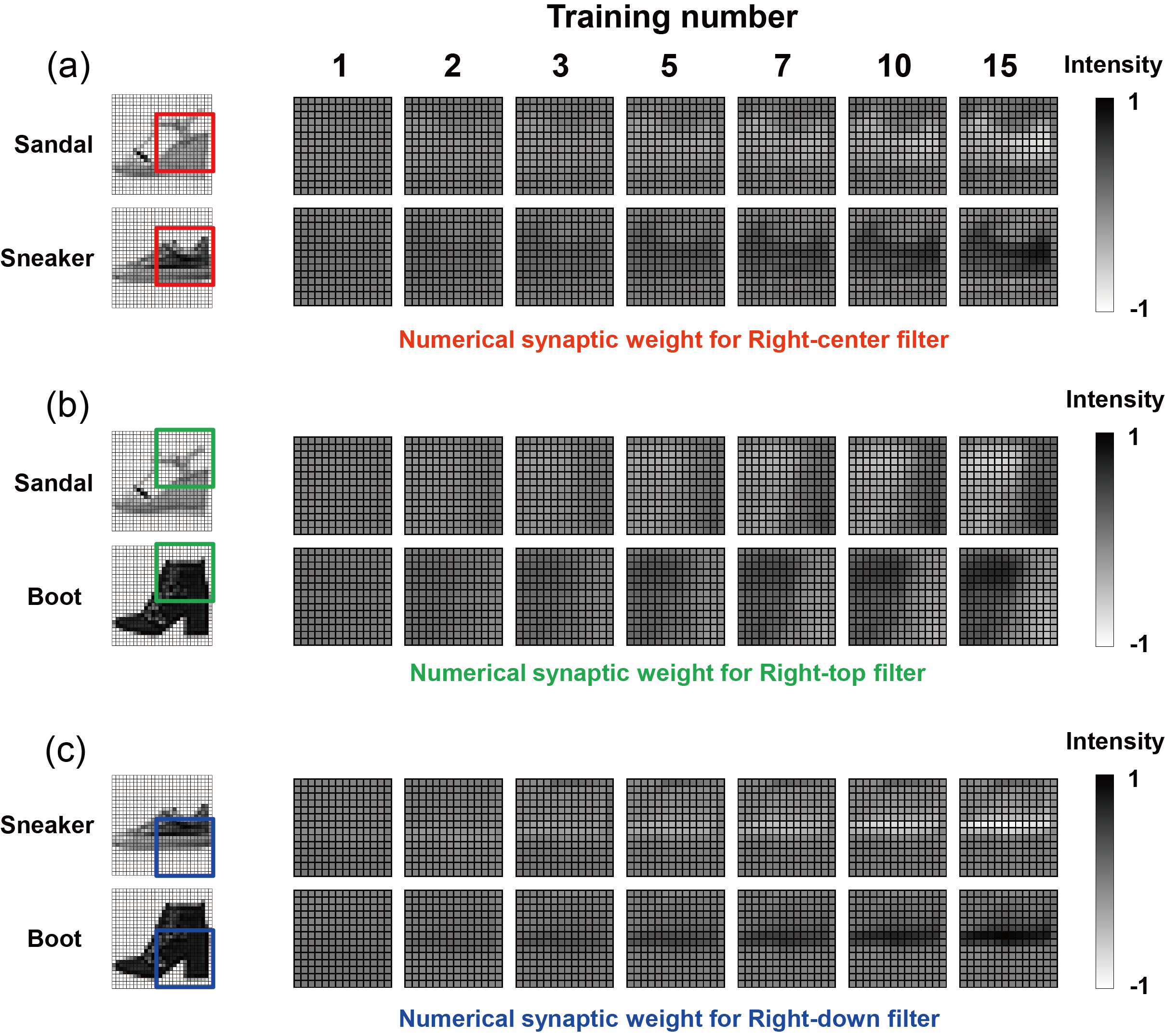
**

**Figure S5.** Reshaped 14 × 14 contour images of the synaptic weight of each filter for numerical simulation with filter evaluation at a δ-value of 0.3 during 15 training numbers for the (a) right-center filter, (b) right-top filter, and (c) right-down filter.

**3. Synaptic weight propagation for numerical simulation**

Figures S4 and S5 present the contour images of the synaptic weight for the numerical simulation process excluding device parameters such as LTP/LTD in the entire region and the filter region, respectively. The overall propagation tendency for the numerical simulation was recovered as shown in Fig. 4 and 5 in the main text, except for the smooth variation of the weight value between neighboring pixels by excluding the deviation of the conductance change in the LTP/LTD curve. All of the weight plots in the filter evaluation were obtained using a δ-value of 0.3 for optimized performance.


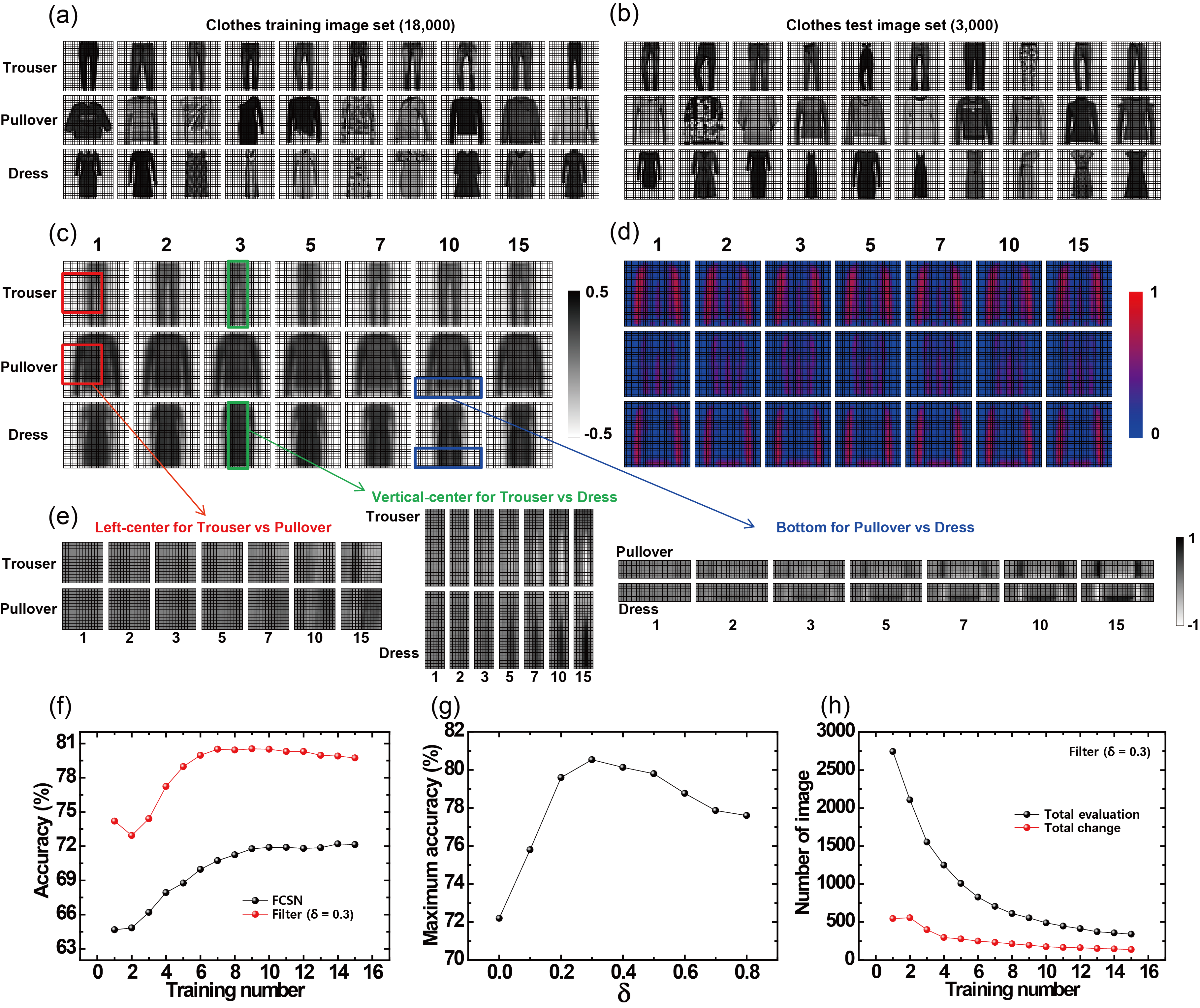


**Figure S6.** (a) 18,000 training images and (b) 3,000 test images for the clothes data set. (c) Average magnitude of the classified training images for each output class at each training epoch in FCSN and (d) absolute difference between the two classes for clothes data set. (e) Reshaped contour images of the synaptic weight of non-square filters at a δ-value of 0.3 during 15 training numbers. (f) Evolution of inference accuracy at the training epoch, (g) maximum accuracy for different δ-values, and (h) number of filter-evaluated test images of the simulation for the clothes data set.

**4. Generalization for other data set with non-square filter evaluation**

The filter-evaluated simulation for run-off election-based decision can be generalized to more complex other data set beyond the shoe data set. Figure S6a-b show the 18,000 training and 3,000 test images for the clothes data set consisting of ‘Trouser’, ‘Pullover’, and ‘Dress’ for each output neuron. The operational principle of the filter evaluation is identical to the case for shoe data set, in which the common feature map was extracted in FCSN for each output class during the training process (Fig. S6c) and the absolute difference contour map between the two output classes was also obtained as shown in Fig. S6d. Because the distinguishable region (red pixel in Fig. S6d) showed non-square trace in general, we also established the filter shape as non-square type (Fig. S6e) as Left-center filter (red box) for Trouser-Pullover, Vertical-center filter (green box) for Trouser-Dress, and Bottom filter (blue box) for Pullover-Dress. Figure S6f shows the inference accuracy of the filter-evaluated numerical simulation for the clothes data set, where the improvement of inference performance was significantly observed at a δ-value of 0.3 (red line) compared with the result for the FCSN (black line). The effect of δ-value variation for the filter evaluation performance is presented in Fig. S6g, in which the maximum accuracy was obtained at a δ-value of 0.3 as ~80.53 % compared to the result for the FCSN as ~72.20 %. As shown in Fig. S6h, the tendency for the number of evaluated clothes test images showed similar decreasing and saturation behavior during the training process in the same manner as in shoe data set.

**References**

S1. Zhao, J., Zhang, M., Wan, S., Yang, Z. & Hwang, C. S. Highly Flexible Resistive Switching Memory Based on the Electronic Switching Mechanism in the Al/TiO2/Al/Polyimide Structure. *ACS Appl. Mater. Interfaces* **10**, 1828 (2018).

S2. Kim, S., Choi, B., Lim, M., Yoon, J., Lee, J., Kim, H.-D. & Choi, S.-J. Pattern Recognition Using Carbon Nanotube Synaptic Transistors with an Adjustable Weight Update Protocol. *ACS Nano* **11**, 2814 (2017).

S3. Burr, G. W., Shelby, R. M., Sidler, S., Nolfo, C., Jang, J., Boybat, I., Shenoy, R. S., Narayanan, P., Virwani, K., Giacometti, E. U., Kurdi, B. N. & Hwang, H. Experimental Demonstration and Tolerancing of a Large-Scale Neural Network (165000 Synapses) Using Phase-Change Memory as the Synaptic Weight Element. *IEEE Trans. Electron Devices* **62**, 3498 (2015).
